# Knocking Out the Gene *RLS1* Induces Hypersensitivity to Oxidative Stress and Premature Leaf Senescence in Rice

**DOI:** 10.3390/ijms19102853

**Published:** 2018-09-20

**Authors:** Guang Chen, Chao Wu, Lei He, Zhennan Qiu, Sen Zhang, Yu Zhang, Longbiao Guo, Dali Zeng, Jiang Hu, Deyong Ren, Qian Qian, Li Zhu

**Affiliations:** 1 State Key Laboratory of Rice Biology, China National Rice Research Institute, Hangzhou 310006, China; chenguang0066@126.com (G.C.); shanzhonghonglou@163.com (L.H.); zhnanqiu@126.com (Z.Q.); zs352523192@126.com (S.Z.); zhangyu881005@163.com (Y.Z.); guolongbiao@caas.cn (L.G.); dalizeng@126.com (D.Z.); hujiang588@163.com (J.H.); rendeyongsd@163.com (D.R.); 2 Institute of Horticulture, Zhejiang Academy of Agricultural Sciences, Hangzhou 310021, China; semporna@126.com

**Keywords:** *Oryza sativa*, oxidative stress response, leaf senescence, reactive oxygen species (ROS), ubiquitin-conjugating enzyme, autophagy

## Abstract

Improving a plant’s level of tolerance to oxidative stress can frequently also enhance its tolerance to several other abiotic stresses. Here, a screen of a *japonica* type rice T-DNA insertion mutant library identified a highly oxidative stress-sensitive mutant. The line exhibited premature leaf senescence, starting at the three-leaf stage, and the symptoms were particularly severe from the five-leaf stage onwards. The leaves progressively lost chlorophyll, suffered protein degradation and were compromised with respect to their photosynthetic activity; their leaf mesophyll and bulliform cells became shrunken, and several senescence-associated genes (*SAG*s), senescence-associated transcription factor genes (*SATF*s) and autophagy-related genes (*ATG*s) were progressively up-regulated. The product of the gene inactivated by the mutation, identified via positional cloning, was putatively a ubiquitin-conjugating enzyme. The gene was denoted here as *RLS1* (reactive oxygen species-sensitive leaf senescence1). The phenotype of plants in which *RLS1* was knocked down using RNA interference was comparable to that of the *rls1* mutant. A comparative analysis of the knock-out line and the wild type leaves showed that the former accumulated more hydrogen peroxide and more malondialdehyde, expressed a heightened level of superoxide dismutase activity and a decreased level of catalase activity, and exhibited an altered transcriptional profile with respect to several *SAG*s, *SATF*s and *ATG*s, and that these effects were magnified when the plants were exposed to oxidative stress. The product of *RLS1* is presumed to be a critical component of the rice oxidative stress response and is involved in ROS (reactive oxygen species)-mediated leaf senescence.

## 1. Introduction

Plants can be exposed to several abiotic stress factors over the course of their life cycle. One of the primary responses to these stresses is to accumulate various reactive oxygen species (ROS), which are used by the plant for signaling [1,2]. A limited, controlled rise in cellular ROS content is required for several beneficial cellular responses to stress to proceed, but excessive levels are cytotoxic, inflicting oxidative damage on membranes, proteins, RNA and DNA, and *in extremis* leading to irreversible cellular damage and even cell death [1,3,4]. Plant cells can neutralize ROS by deploying several enzymes (superoxide dismutase (SOD), catalase (CAT), peroxidase (POD) and ascorbate peroxidase (APX)) and anti-oxidants (ascorbic acid and reduced glutathione) [1,3,4].

Premature senescence of the leaf can be triggered both by various external factors, notably drought, salinity, shading and disease, and by endogenous factors such as the cellular sugar content and the level of some phytohormones [5,6,7]. A burst in ROS production is a common cause of leaf senescence [8,9,10,11]. In rice, the over-expression of *OsSIK2,* a gene which encodes an S-domain receptor-like kinase, has the effect of delaying leaf senescence through its enhancement of peroxidase activity [8]. The inactivation of *OsUAP1* (which encodes a UDP-N-acetylglucosamine pyrophosphorylase) raises the cellular level of ROS, thereby accelerating leaf senescence [9]. Similarly, a mutation to the gene *OsNaPRT1* results in the accumulation of hydrogen peroxide (H_2_O_2_) and consequently to premature leaf senescence [12]. Finally, disrupting *OsMTS1*, a gene which encodes a methyltransferase, triggers leaf senescence because of its suppression of the production of the free radical scavenging compound melatonin [11].

Oxidative stress is frequently a side effect of other abiotic stresses [13,14], so that an improvement in a plant’s level of tolerance to oxidative stress has the potential to support its ability to combat other stresses [15,16]. Here, the aim was to identify rice genes associated with the oxidative stress response and/or with tolerance to abiotic stress by subjecting a collection of T-DNA insertion mutants to oxidative stress. The experiment, conducted using a convenient and stable hydroponics system, identified a mutant which was highly sensitive to stress. Positional cloning of the mutated gene showed that its product was a ubiquitin-conjugating enzyme, likely involved in the regulation of both the oxidative stress response and in ROS-mediated leaf senescence.

## 2. Results

### 2.1. Isolation of a Rice Oxidative Stress-Sensitive Mutant

**Figure 1:**
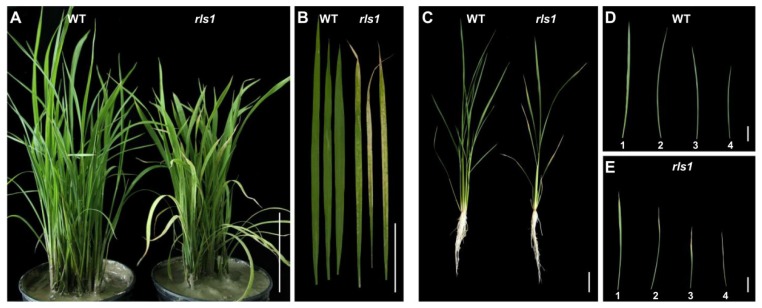
The phenotype of WT (wild type) and *rls1* plants. (**A**) Soil-grown seedlings at the five-leaf stage; (**B**) the appearance of fully expanded seedling leaves; (**C**) hydroponics-grown 35 day old plants at the tillering stage; (**D**,**E**) the appearance of the first, second, third and fourth fully expanded leaf (counting from the apex to the base of the main tiller) of 35 day old (**D**) WT and (**E**) *rls1* mutant plants. Bars in (**A**,**C**): 10 cm, in (**B**,**D**,**E**): 5 cm.

The screen of the T-DNA insertion mutant collection revealed a line which was hypersensitive to oxidative stress. When exposed 60 mM H_2_O_2_ for two days, the plants wilted, and their leaves began to exhibit chlorosis; in contrast, WT (wild type) plants suffered only minor wilting [17]. Prior to the stress treatment, there was no morphological difference between the leaves of the mutant and those of WT seedlings. By the time the plants reached the five-leaf stage, the tips of their mature leaves had become necrotic (Figure 1A,B), while their newly emerging leaves were no different to WT ones (Figure 1A). The necrosis began at the tips of the fully expanded leaf, then spread into the leaf blade as the plants developed (Figure 1C–E). The corresponding leaves formed by WT plants were non-necrotic (Figure 1D). Based on its oxidative stress sensitivity and leaf phenotype, the mutant was designated *rls1* (for ROS-sensitive leaf senescence).

### 2.2. The Genetic Basis of the rls1 Mutation and Its Lack of Association with a T-DNA Insertion

A segregation analysis of 300 F_2_ progeny bred from the cross *rls1* × WT showed that 212 individuals exhibited the WT phenotype and 88 the *rls1* phenotype, fitting the monogenic ratio of 3:1 (χ^2^ = 2.78 < χ^2^_0.05_ = 3.84). Since the mutant was selected from a T-DNA insertion library, the expectation was that the mutant phenotype would co-segregate with the presence of a T-DNA sequence. Based on the presence of a T-DNA as inferred from the outcome of a PCR targeting the gene *hpt*, this result was not obtained. As the mutation was therefore evidently not induced by a T-DNA insertion event, a positional cloning strategy was used to identify the gene compromised in the *rls1* mutant.

### 2.3. Positional Cloning of the Gene Underlying the rls1 Mutation

**Figure 2:**
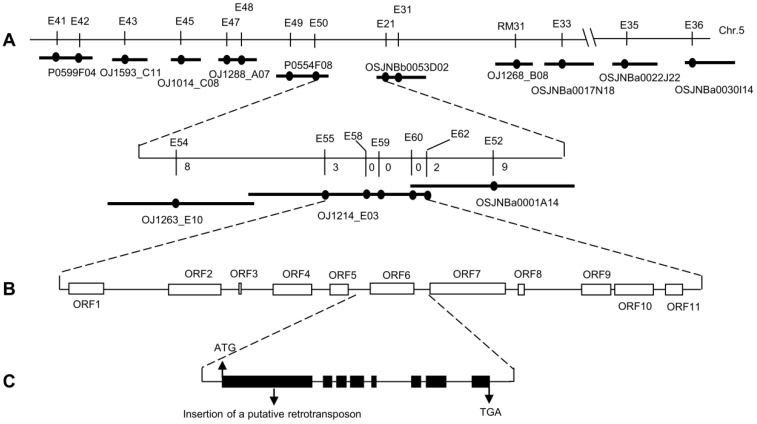
The positional cloning of *RLS1*. (**A**) *RLS1* was first found to be linked to the SSR (simple sequence repeat) marker RM31 on chromosome 5; fine mapping narrowed its location to a 67.5 kb genomic DNA segment flanked by STS (sequence tagged site) markers E55 and E62; (**B**) the critical genomic region’s sequence includes 11 open reading frames; (**C**) the structure of the *RLS1* candidate gene LOC_Os05g48390. Both the start (ATG) and stop (TGA) codons are indicated. Black boxes indicate coding sequence. The site of the Tos17 insertion in the mutant’s *RLS1* sequence is shown.

A coarse level linkage map, based on 121 F_2_ progeny constructed from the cross *rls1* × cultivar (cv.) Longtepu, placed the mutated gene (designated *RLS1*) on chromosome 5, near the SSR locus RM31 (Figure 2A). A higher resolution map was then generated by genotyping a large set of F_2_ individuals which all displayed the *rls1* phenotype, using 20 additional SSR (simple sequence repeat) and STS (sequence tagged site) markers mapping to the candidate region (Table S1); the outcome of this genotypic analysis was to narrow the site of *RLS1* to a 67.5 kb interval flanked by the STS markers E55 and E62, both of which lie on the rice BAC (bacterial artificial chromosome) clone OJ1214_E03 (Figure 2A). The interval harbors eleven open reading frames (ORFs) (http://www.gramene.org/Oryza_sativa/Location/View?db=core&h=BLAST_NEW%3ABLA_1SNEGPAZJ%21%21&r=5%3A27700995-27768494), nine of which are matched by a full-length cDNA (Figure 2B). Re-sequencing in *rls1* failed to identify any mutations in ten of the eleven putative genes. However, for the eleventh gene, the primer pair FE14P1/FE14P22 (Table S1) amplified a 705 bp fragment from a WT template but produced no amplicon from a *rls1* template. The possibility that the *rls1* mutation resulted from a Tos17 insertion event was tested using a thermal asymmetric interlaced PCR (TAIL-PCR) assay. The analysis showed that the *rls1* mutant harbored a 4.1 kb stretch of Tos17 sequence derived from a site on chromosome 7 and transposed to the *RLS1* exon (on chromosome 5) during tissue culture (Figure 2C).

### 2.4. The Phenotype of RLS1-RNAi Transgenic Plants

**Figure 3:**
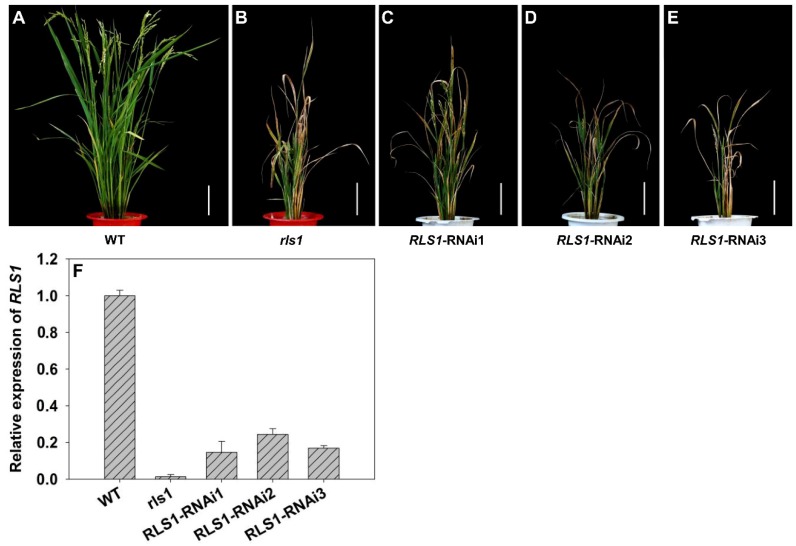
Comparison of WT, *rls1* and *RLS1*-RNAi plants at the reproductive stage. (**A**,**B**) The performance of (**A**) WT, (**B**) *rls1* plants grown in paddy soil to the heading stage (bars: 15 cm); (**C**–**E**) the phenotype of an *RLS1*-RNAi transgenic plant at the heading stage (bars: 15 cm); (**F**) the abundance of *RLS1* transcript in the fully expanded leaf of WT, *rls1* and *RLS1*-RNAi. For normalization purposes, the transcript abundance of *RLS1* in WT was set to 1. Values are expressed in the form mean ± SE (*n* = 3).

The RNAi construct introduced into WT cv. Nipponbare designed to knock down *RLS1* comprised a 447 bp segment of the coding region. The effect of the transgene was to substantially reduce the abundance of *RLS1* transcript in the leaves (Figure 3F). The phenotype of plants carrying the transgene was similar to that of the *rls1* mutant (Figure 3A–E). Transgenic plants carrying an empty vector were phenotypically indistinguishable from WT [18].

### 2.5. Microstructural Analysis of the rls1 Mutant Leaf

**Figure 4:**
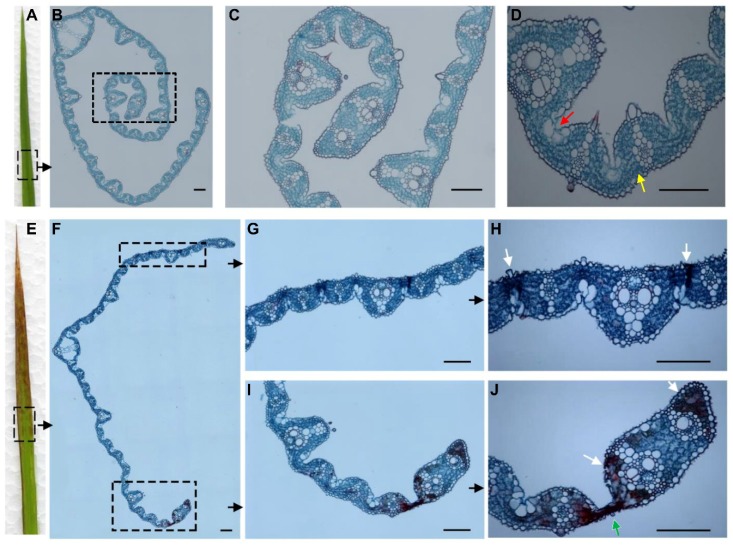
The microstructure of the leaf of WT and *rls1* plants. (**A**–**D**) WT leaves feature normal mesophyll (yellow arrow) and bulliform (red arrow) cells; (**E**–**J**) The bulliform cells in the leaf margin of *rls1* plants (green arrow) appear shriveled and atrophied. A portion of the mutant’s mesophyll cells appear atrophied and dead (white arrows). Bars: 100 µm.

The microstructure of freshly harvested, fully expanded leaves was investigated by light microscopy. A comparison of the morphology of the mesophyll and bulliform cells in the WT and *rls1* leaf (Figure 4) suggested that the premature leaf senescence displayed by the mutant was largely a result of their atrophy.

### 2.6. The Growth Response of the rls1 Mutant to Oxidative Stress

**Figure 5:**
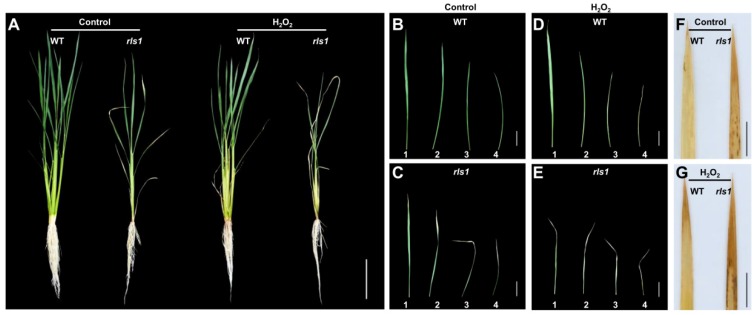
The performance of WT and *rls1* plants in response to oxidative stress imposed by exposure to 60 mM H_2_O_2_ for two days. (**A**) *rls1* and WT plants grown under either non-stressed or stressed conditions. Bar: 10 cm; (**B**–**E**) the appearance of the first, second, third and fourth fully expanded leaf (counting from the apex to the base of the main tiller) of (**B**,**D**) WT, (**C**,**E**) *rls1* plants grown (**B**,**C**) in the absence and (**D**,**E**) in the presence of oxidative stress. Bars: 5 cm. (**F**,**G**) Histochemical detection (DAB staining) of H_2_O_2_ in the second fully expanded leaf of plants grown under (**F**) non-stressed, (**G**) stressed conditions. Bars: 2 cm.

Under non-stressed conditions, the mutant plants’ growth was slower than that of WT plants, resulting in the development of a smaller number of tillers in six-week-old plants (Figure 5A). Leaf tip necrosis occurred first in the lower leaves, spreading later to the younger leaves (Figure 5B,C). When exposed to 60 mM H_2_O_2_, *rls1* plants suffered from extensive wilting, while their leaves rapidly developed chlorosis and withered from their tip to their base (Figure 5E). The stress treatment promoted leaf senescence in both WT and *rls1* plants (Figure 5B–E), but both the rate of development of the symptoms and their severity were lower in the WT leaf than in the corresponding *rls1* leaf (Figure 5D,E). An assay used to quantify H_2_O_2_ in the *rls1* leaf, based on 3,3′-diaminobenzidine (DAB), showed that the regions which accepted the stain coincided with those which became chlorotic; *rls1* leaves became heavily stained, whereas in WT plants subjected to oxidative stress, only the leaf tips accepted the stain (Figure 5F,G).

### 2.7. The Physiological Indicators of Premature Leaf Senescence in the rls1 Mutant

**Figure 6:**
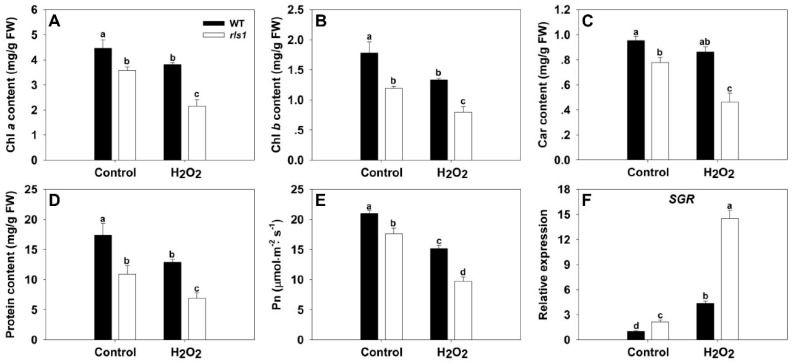
The physiological effect of oxidative stress on WT and *rls1* plants. (**A**–**C**) The content of photosynthetic pigments in the full expanded leaf: (**A**) chlorophyll *a*; (**B**) chlorophyll *b*; (**C**) carotenoid; (**D**) protein content; (**E**) the net photosynthetic rate of the leaf; (**F**) the abundance of *SGR* (senescence-induced STAY GREEN) transcript. For normalization purposes, the transcript abundance of *SGR* in non-stressed WT was set to 1. Values expressed in the form mean ± SE ((**A**–**E**): *n* = 5, F: *n* = 3). Significant differences between means (*p* < 0.05) indicated by different letters. FW: fresh weight.

Chlorophyll loss and protein degradation are widely taken as markers of leaf senescence [19,20]. A comparison between the leaves of *rls1* and WT plants grown under non-stressed conditions showed that the content of both the major photosynthetic pigments and total protein in the former was uniformly lower than that in the latter (Figure 6A–D). In plants exposed to H_2_O_2_ treatment, both the leaf chlorophyll and protein content was reduced not just in *rls1* plants, but also in WT ones, but the extent of the decline was greater in the mutant. Measurement of the net photosynthetic rate (Pn) of leaves developed by non-stressed plants also revealed a significant difference between mutant and WT plants; in stressed plants, the difference in Pn between the mutant and the WT was magnified (Figure 6E). Quantification of the transcript abundance of *SGR* (senescence-induced STAY GREEN), the product of which regulates chlorophyll degradation [9], showed that it was higher in *rls1* leaves than in WT leaves developed under non-stressed conditions; the difference was even larger in stressed leaves (Figure 6F).

### 2.8. The rls1 Mutant Exhibits an Altered Profile of ROS (Reactive Oxygen Species) and MDA (Malondialdehyde) Accumulation, Accompanied by Changes in Anti-Oxidative Enzyme Activity

**Figure 7:**
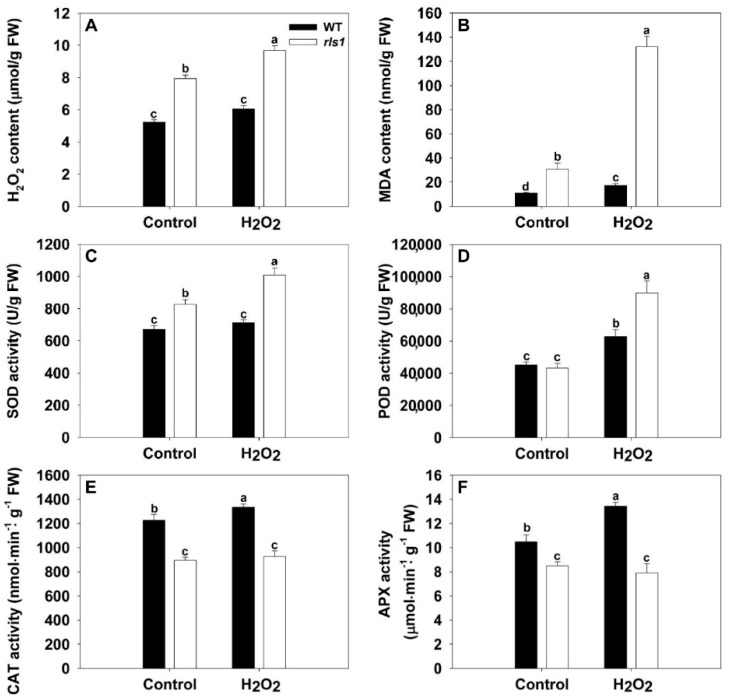
The biochemical response of WT and *rls1* plants to exposure to oxidative stress. The content in the leaf of (**A**) H_2_O_2_; (**B**) MDA; (**C**–**F**) the activity of the anti-oxidant enzymes (**C**) SOD (superoxide dismutase); (**D**) POD (peroxidase); (**E**) CAT (catalase); (**F**) APX (ascorbate peroxidase). Values expressed in the form mean ± SE (*n* = 5). Significant differences between means (*p* < 0.05) indicated by different letters. FW: fresh weight.

The H_2_O_2_ content of *rls1* leaves was 50% higher than that of WT leaves developed by non-stressed plants, increasing to 60% in stressed ones (Figure 7A). The MDA (malondialdehyde) content of *rls1* leaves was 2.8-fold that of WT leaves of plants raised under non-stressful conditions, with the difference becoming even greater when stressed leaves were compared (Figure 7B). Prior to the H_2_O_2_ treatment, the level of SOD activity in *rls1* leaves was significantly higher than that in WT leaves (Figure 7C), that of POD did not differ between *rls1* and WT leaves (Figure 7D), while that of both CAT and APX were significantly lower (Figure 7E,F). SOD activity was promoted in *rls1* plants subjected to oxidative stress, while there was no significant difference in SOD activity between stressed and non-stressed WT plants (Figure 7C). The activities of POD, CAT and APX all increased significantly in WT in response to the stress (Figure 7D–F); the increase was even more marked for POD activity in the mutant, resulting in it reaching a significantly higher level in the mutant (Figure 7D). Both CAT and APX activity was unaffected by the stress in the mutant, with the result that both remained lower than in WT (Figure 7E,F).

### 2.9. SAGs (Senescence-Associated Genes), SATFs(Senescence-Associated Transcription Factor Genes) and ATGs (Autophagy-Related Genes) Were All Up-Regulated in the rls1 Mutant

**Figure 8:**
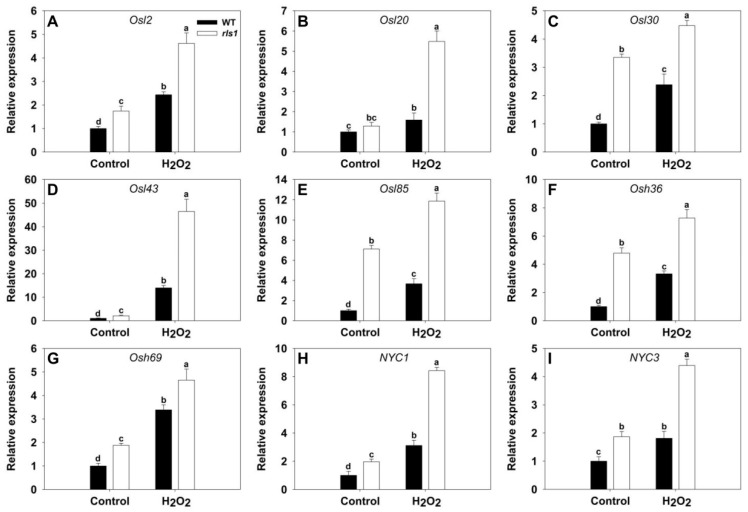
The transcriptional response of SAGs (senescence-associated genes) in WT and *rls1* plants subjected to oxidative stress. (**A**) *Osl2*; (**B**) *Osl20*; (**C**) *Osl30*; (**D**) *Osl43*; (**E**) *Osl85*; (**F**) *Osh36*; (**G**) *Osh69*; (**H**) *NYC1*; (**I**) *NYC3*. For normalization purposes, the transcript abundance of each *SAG* in non-stressed WT was set to 1. Values expressed in the form mean ± SE (*n* = 3). Significant differences between means (*p* < 0.05) indicated by different letters.

Based on qRT-PCR assays, under non-stressed conditions, most of the genes tested were transcribed more strongly in *rls1* than in WT; the exception was *Osl20*, which was transcribed at a similar level in both lines (Figure 8). The genes were, as anticipated, all up-regulated by the imposition of oxidative stress; the increase in transcript abundance was more marked in *rls1* than in WT: thus the fold differences between *rls1* and WT leaves of *Osl2*, *Osl20*, *Osl30*, *Osl43*, *Osl85*, *Osh36*, *Osh69*, *NYC1* and *NYC3* transcript abundance were, respectively, 1.90, 3.45, 1.88, 3.32, 3.25, 2.19, 1.38, 2.70 and 2.43. 

**Figure 9:**
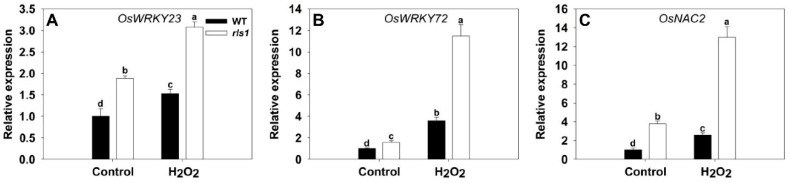
The transcriptional response of SATFs (senescence-associated transcription factor genes) in WT and *rls1* plants subjected to oxidative stress. (**A**) *OsWRKY23*; (**B**) *OsWRKY72*; (**C**) *OsNAC2.* For normalization purposes, the transcript abundance of each *SATF* in non-stressed WT was set to 1. Values expressed in the form mean ± SE (*n* = 3). Significant differences between means (*p* < 0.05) indicated by different letters.

An investigation as to whether any transcription factors contributed to the premature senescence syndrome of *rls1* showed that *OsWRKY23*, *OsWRKY72* and *OsNAC2* were all more strongly transcribed in *rls1* than in WT plants grown under non-stressed conditions (Figure 9). Exposure to H_2_O_2_ up-regulated all three genes, more so in *rls1* plants than in WT ones. The behavior of *OsWRKY72* was especially notable: in the mutant, this gene was only weakly transcribed under non-stressed conditions, but in plants challenged by H_2_O_2_, the abundance of its transcript rose by about five-fold. 

**Figure 10:**
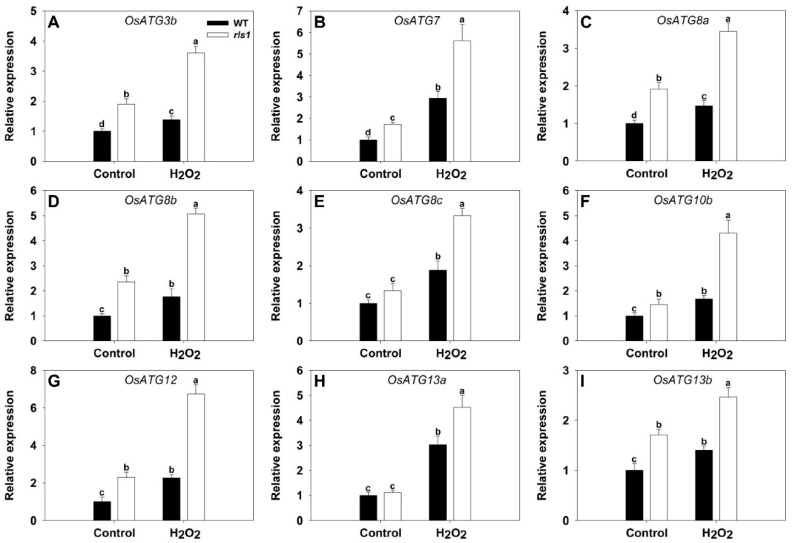
The transcriptional response of ATGs (autophagy-related genes) in WT and *rls1* plants subjected to oxidative stress. (**A**) *OsATG3b*; (**B**) *OsATG7*; (**C**) *OsATG8a*; (**D**) *OsATG8b*; (**E**) *OsATG8c*; (**F**) *OsATG10b*; (**G**) *OsATG12*; (**H**) *OsATG13a*; (**I**) *OsATG13b*. For normalization purposes, the transcript abundance of each *ATG* in non-stressed WT was set to 1. Values expressed in the form mean ± SE (*n* = 3). Significant differences between means (*p* < 0.05) indicated by different letters.

The transcript abundance of all nine *ATG*s assessed differed between *rls1* and WT plants grown in the presence of stress, but these differences disappeared for both *OsATG8c* and *OsATG13a* in non-stressed plants (Figure 10). The stress up-regulated each of the nine genes, with the extent of induction being lower in WT than in *rls1*; the range in the relative transcript abundance was from 1.49-fold (*OsATG13a*) to 2.98 (*OsATG12*).

## 3. Discussion

### 3.1. Functional Inactivation of RLS1 Enhances Sensitivity to Oxidative Stress

The screen of a set of T-DNA insertion mutants, based on their response to oxidative stress, revealed several hypersensitive lines, one of which—*rls1*—displayed a particularly severe degree of wilting and foliar chlorosis (Figure 5). The gene responsible for this mutant’s phenotype was shown via positional cloning to be LOC_Os05g48390 (Figure 2), here given the designation *RLS1*. According to the Rice Genome Annotation Project (http://rice.plantbiology.msu.edu/cgi-bin/gbrowse/rice/), this gene encodes a ubiquitin-conjugating enzyme E2. Ubiquitin-conjugating enzymes have been reported to be involved in plant tolerance to a range of abiotic stresses: for example, in tomato, the transcription of *UBC1* is enhanced by exposure to both heat shock and cadmium chloride, suggesting the involvement of its product in the degradation of abnormal proteins induced by these abiotic stresses [21]. In *Arabidopsis* *thaliana*, the product of *AtUBC32* contributes to brassinosteroid-mediated salinity tolerance [22] and the over-expression of the soybean gene *GmUBC2* has been shown to enhance both drought and salinity tolerance, suggesting the involvement of its product in ion homeostasis, osmolyte synthesis and the oxidative stress response [23]; similarly, the constitutive expression of the groundnut (*Arachis hypogaea*) gene *UBC2* improves the tolerance of *A. thaliana* to drought [24], while that of the mung bean (*Vigna radiata*) gene *UBC1* enhances its osmotic stress tolerance [25]. Furthermore, the over-expression of tobacco *UBC1* promotes the plant’s tolerance of cadmium stress [26]. The present report is the first demonstration that alterations in the expression of a *UBC* gene can affect the oxidative stress response in rice.

### 3.2. The Inactivation of RLS1 Induces Premature Leaf Senescence

The most visible physiological change which takes place during leaf senescence is the gradual loss of chlorophyll [7,27,28]; this was also the case for the premature senescence experienced by *rls1* (Figure 6A–C). The development of senescence in *rls1* was accompanied by the up-regulation of *SGR* (Figure 6F); the product of this gene has been suggested to actively regulate chlorophyll degradation [9]. Proteolysis is also well recognized as an indicator of senescence [20], so, as predicted, the protein content of the *rls1* leaves was lower than that of WT leaves (Figure 6D). A reduction in Pn is yet another consequence of leaf senescence [7,11,28], and was borne out by the observation that the Pn in the leaves of *rls1* was lower than in WT leaves (Figure 6E). The onset and progression of senescence are accompanied by numerous transcriptomic changes, among which is the up-regulation of *SAG*s [9,20]. Consistent with this, the abundance of *SAG* transcripts proved to be higher in *rls1* than in WT (Figure 8). The same applied for the *SATF*s *OsWRKY23*, *OsWRKY72* and *OsNAC2*, all of which are known to be up-regulated during leaf senescence [9,20,29]: they were similarly each transcribed more strongly in *rls1* than in WT (Figure 9). The autophagy genes *OsATG4b*, *OsATG8a* and *OsATG18b* have also all been shown to be up-regulated during leaf senescence [20], and again they were all transcribed more abundantly in *rls1* than in WT (Figure 10). The stress had an inductive effect on these genes, particularly so in *rls1,* consistent with the more rapid and widespread senescence experienced by the mutant (Figure 5). These outcomes support the notion that the premature leaf senescence displayed by *rls1* is genetically programmed. As the loss-of-function of *RLS1* resulted in premature leaf senescence, the likelihood is that in WT plants, the product of *RLS1* acts to regulate this process.

### 3.3. ROS Are Instrumental in the Development of the Premature Leaf Senescence of rls1

The process of senescence is promoted by the accumulation of ROS [9,11], and especially that of H_2_O_2_ [20,30,31]. A feature of the *rls1* mutant was its rapid accumulation of H_2_O_2_ (Figure 5 and Figure 7), which was accompanied not just by premature leaf senescence, but also by its reduced tolerance of oxidative stress (Figure 5), by its loss of photosynthetic pigments, by its fall in Pn (Figure 6) and by the induction of a range of senescence-associated genes (Figure 6, Figure 8, Figure 9 and Figure 10). The notion that the heightened content of ROS in the leaf of the *rls1* mutant is, at least in part, responsible for the premature leaf senescence syndrome and the heightened sensitivity to oxidative stress was consistent with the observation that H_2_O_2_ exposure raised the leaf ROS content, thereby accelerating and exacerbating the syndrome (Figure 5 and Figure 6). The tissue content of MDA, a product of membrane lipid peroxidation, is widely used as a marker for oxidative stress-induced cellular damage [27,32]. The *rls1* mutant developed a significantly higher level of MDA in its leaves than did WT (Figure 7B). The conclusion is that the over-production of and/or the less effective scavenging capacity of ROS is the most likely reason for the greater degree of lipid peroxidation which occurs in the leaves of *rls1* plants exposed to oxidative stress (Figure 7A,B). Notably, in the mutant’s leaf, CAT activity was lower than in the WT leaf (Figure 7E). Upon exposure to H_2_O_2_, several known *SAG*s were up-regulated in both the mutant and the WT, with a more marked increase in transcript abundance observed in the former line (Figure 8), mirroring the stress-induced up-regulation of the three *SAG*s *Osl20*, *Osl85* and *Osh69* [20]. The nine *ATG*s, which were all up-regulated by the stress, included *OsATG8a* and *OsATG10b* (Figure 10). *OsATG8a* is homolog of the *A.* *thaliana* gene *AtATG8a*, which also responds transcriptionally to H_2_O_2_ treatment [20]. A loss-of-function *OsATG10b* mutant has been shown to be particularly sensitive to treatment with the ROS-inducing reagent methyl viologen, and an intact version of the gene appears to be important for plant survival in the face of oxidative stress [33]. Several transcription factors have been revealed to mediate ROS-regulated leaf senescence [34]. Here, the H_2_O_2_ treatment promoted the transcription of the genes encoding several transcription factors, in particular *OsWRKY72* (Figure 9). The latter gene is known to be inducible by treatments with abscisic acid, salinity, ozone and H_2_O_2_ [20,35,36]. The WRKY domains in OsWRKY72 and AtWRKY53 share >60% identity at the amino acid level; as AtWRKY53 is known to mediate H_2_O_2_-induced leaf senescence [37], it is plausible that OsWRKY72 does the same in rice. The nature of any dependence of the *SAG*s and *ATG*s identified here on specific transcription factor(s) is unknown, but it has been established that OsNAP influences leaf senescence by directly targeting *SAG*s [38]. Similarly, in *A.* *thaliana*, AtWRKY33 interacts with AtATG18a [39]. The likelihood is therefore that transcription factors, putatively members of the NAC and/or WRKY family, are involved in ROS-mediated senescence in the *rls1* mutant.

In conclusion, the phenotype of the selected highly oxidative stress-sensitive mutant featured the development of necrosis in the leaf tips starting at the five-leaf stage, after which the whole leaf senesced rapidly. The gene which had been inactivated in the mutant was shown to encode a ubiquitin-conjugating enzyme. A series of functional analyses, based on a range of physiological, biochemical, and molecular assays, was able to demonstrate that the functional inactivation of *RLS1* promoted hypersensitivity to oxidative stress and induced premature leaf senescence, both of which resulted from the accumulation of ROS. The suggestion is that the product of *RLS1* is required to regulate the response to oxidative stress and ROS-mediated leaf senescence in rice.

## 4. Materials and Methods 

### 4.1. Plant Materials and Growing Conditions

A collection of T-DNA insertion mutant lines, developed in the *japonica* type cv. Nipponbare [40], was grown under hydroponics in a growth chamber delivering a 14 h photoperiod, with the temperature set to 30 °C during the lit period and 22 °C during the dark period, and a relative humidity level maintained at ~70%. The culture solution, formulated as described by Chen et al. [41], was replaced every two days. Following the procedure given by Xiong et al. [42], ten-day old seedlings of uniform size were transferred into the same solution supplemented with 60 mM H_2_O_2_ for two days, at which point the extent of necrotic and chlorotic damage was determined. The experiment consisted of three replicates, each involving 16 seedlings per line. To compare the response of the selected mutant line and WT to oxidative stress, seedlings were grown in the above culture solution for six weeks before being transferred to the same solution containing 60 mM H_2_O_2_ for two days. Fully expanded leaf blades were harvested, weighed, and then used for a variety of physiological and molecular assays. This experiment was also run in triplicate, each replicate comprising five plants of each line. In a soil-based experiment conducted to compare the performance of WT, the mutant line and an RNAi knock-down line, seedlings were grown in a greenhouse up to the five-leaf stage, after which single seedlings were transplanted into a pot filled with 10 kg air-dried loam soil, following Chen et al. [43]. Various growth parameters were assessed, both at the time of transplantation and at the heading stage. This experiment was also run in triplicate, each replicate comprising five plants per each line. The same greenhouse environment was used for the hydroponics and the soil-based experiments. An F_2_ population, bred from a cross between the selected T-DNA mutant and the *indica* type cv. Longtepu was grown during the normal growing season in an experimental field at the China National Rice Research Institute (Hangzhou, China).

### 4.2. Isolation of the Target Gene

The initial strategy for gene isolation was to search for the presence of a T-DNA sequence in the selected mutant, since the mutation was assumed to have derived from a T-DNA insertion event. The PCR screen used for this purpose was based on a 600 bp fragment of *hpt*, assayed using the primer pair HPT1/2 (respectively, 5′-GTTTATCGGCACTTTGCATCG-3′ and 5′-GGAGCATATACGCCCGGAGT-3′). When this approach failed, a positional cloning strategy was adopted. Here, an initial coarse level genetic map, based on a genome-wide set of SSR and STS markers, was constructed by segregational analysis in a set of 121 F_2_ progeny bred from a cross between the selected mutant and cv. Longtepu. Based on the approximate chromosomal location determined in this way, additional markers (both STS and SSR) known to lie within the critical region were then used to fine-map the gene responsible for the mutation in a set of 1545 F_2_ progeny exhibiting the mutant phenotype. These additional markers were generated from a comparison between the relevant sequences of the *indica* type cv. 9311 and the *japonica* type cv. Nipponbare. The PCRs used for genotyping employed an initial denaturation of 94 °C/4 min, followed by 35 cycles of 94 °C/30 s, annealing temperature (dependent on the primer pair)/30 s and 72 °C/60 s, with a final extension of 72 °C/10 min. Amplicons were separated by electrophoresis through a 5% agarose gel. The sequences of the primers used are listed in Table S1.

### 4.3. Sequencing Analysis of Candidate Region

The genomic region harboring the target gene, as defined by fine mapping, contained several putative genes; each of these was targeted by a PCR assay, which was conducted on genomic DNA extracted from plants expressing the mutant phenotype. The resulting amplicons were gel-purified and submitted to the Shanghai Sunny Biotechnology Co. Ltd. (Shanghai, China) for sequencing. The plants used for this purpose included the original mutant line and an F_2_ segregant from the mapping cross. TAIL-PCR was used to isolate genomic sequences flanking the insertions in the transgenic plants [44]. The genomic sequence flanking the Tos17 insertion on the left side was amplified using the primers ls4F, ls41F and ls42F, and that on the right-side primers ls4R, ls421R and ls422R (Table S1). The degenerate primers required were those described by Zhu et al. [45]. The resulting amplicons were sequenced by the Shanghai Sunny Biotechnology Co. Ltd. (www.sunny-biotech.com, Shanghai, China). The sequences of the primers used for sequencing are listed in Table S1.

### 4.4. RNAi Knock-Down Vector Construction

A 447 bp fragment of the target gene was PCR-amplified, using the primer pair E70rF/R (respectively 5′-CACCTTTACAGTCCGTTCAA-3′ and 5′-GTCAATCATCAAGTCCTCCA-3′), and the amplicon was inserted into the Gateway pENTR/D-TOPO cloning vector [45]. The insert was subsequently transferred into pANDA35HK using the recombinase reaction [46]. Either the pANDA340-E70 (RNAi knock-down) or the pANDA35HK (empty vector control) plasmid was introduced into cv. Nipponbare plants using agroinfection, based on the *Agrobacterium tumefaciens* strain EHA105, following the procedures given by Chen et al. [47].

### 4.5. Quantitative Real Time PCR (qRT-PCR)

The transcriptional effect of knocking down the target gene was tested using qRT-PCR. RNA was extracted from fully expanded leaf blades of plants carrying the RNAi construct and WT. *OsACT1* (LOC_Os03g50885) was chosen as the reference sequence. The primers used to amplify the target sequence were re340F/R (respectively 5′-CAATGGGCTAGTGGTGCGATA-3′ and 5′-GGTCGTCCAGGCTATGAACAA-3′) and those for the reference gene were RRAC2F/R (respectively 5′-GCTATGTACGTCGCCATCCA-3′ and 5′-GGACAGTGTGGCTGACACCAT-3′). The qRT-PCR methodology followed that given by Chen et al. [41]. To compare the effect of oxidative stress on the transcriptome of the mutant and WT, RNA was extracted from fully expanded leaves of plants raised either in the presence or the absence of 60 mM H_2_O_2_. The rice ubiquitin gene *UBQ5* (LOC_Os01g22490) was chosen as the reference sequence for these qRT-PCRs. Relative transcript abundances were calculated following Chen et al. [48]. The sequences of the various primers used in qRT-PCR experiments are listed in Table S2.

### 4.6. Leaf Tissue Morphology

Paraffin sections used for the microscopic analysis of leaf tissue morphology were prepared from freshly harvested, fully expanded leaves of both WT and the selected mutant. Following the procedures described by Liu et al. [49], the samples were held overnight at 4 °C in a solution composed of 10 mL formalin, 5 mL glacial acetic acid and 85 mL 70% ethanol, after which they were dehydrated by passing through an ethanol series. Following their fixation in xylene, the sections were embedded in Paraplast and sectioned into 8 µm slices using a Leica rotary microtome. The sections were finally stained in 0.5% Fast Green FCF and observed under light microscopy.

### 4.7. Biochemical Characterization

For the histochemical-based staining used to detect H_2_O_2_ in the leaf, leaves of WT and the selected mutant plants were processed as described by Zhou et al. [20]. Each experiment was conducted in triplicate. The quantification of photosynthetic pigment was performed following Deng et al. [50]. The method used to quantify soluble protein content was as described by Zhou et al. [20]. Leaf MDA contents were assessed using the protocol given by Chen et al. [51]. To determine the H_2_O_2_ and ROS-associated enzyme content in fully expanded leaves, leaf material was snap-frozen, ground to a powder and stored at −80 °C. Kits purchased from Comin Biotechnology Co., Ltd. (Suzhou, China) were used to quantify the content of H_2_O_2_ and the activity of SOD, POD, CAT and APX, as described by Pan et al. [52].

### 4.8. Photosynthetic Rate

Net photosynthetic rates were measured from plants between 9.00 and 11.00 am using a Li-COR6400 portable photosynthesis system equipped with a LED leaf cuvette (Li-COR, Lincoln, NE, USA), as described by Chen et al. [53]. At least five individuals per line per treatment were measured.

### 4.9. Statistical Analysis

Analyses of variance were carried out using routines implemented in SPSS v10 software (SPSS Inc., Chicago, IL, USA). Different letters appearing over histogram columns are used to indicate statistically differences at *p* < 0.05, as determined by Tukey’s test.

## Supplementary Materials

Supplementary materials can be found at http://www.mdpi.com/1422-0067/19/10/2853/s1.

## References

[1] Choudhury F.K., Rivero R.M., Blumwald E., Mittler R. (2017). Reactive oxygen species, abiotic stress and stress combination. Plant J..

[2] Mittler R. (2017). ROS are good. Trends Plant Sci..

[3] Nasser S., Kemal K., Schenk P.M. (2016). Global plant stress signaling: Reactive oxygen species at the cross-road. Front. Plant Sci..

[4] Zhang H., Liu X.L., Zhang R.X., Yuan H.Y., Wang M.M., Yang H.Y., Ma H.Y., Liu D., Jiang C.J., Liang Z.W. (2017). Root damage under alkaline stress is associated with reactive oxygen species accumulation in rice (*Oryza sativa* L.). Front. Plant Sci..

[5] Van Doorn W.G. (2008). Is the onset of senescence in leaf cells of intact plants due to low or high sugar levels?. J. Exp. Bot..

[6] Schippers J.H.M., Schmidt R., Wagstaff C., Jing H. (2015). Living to die and dying to live: The survival strategy behind leaf senescence. Plant Physiol..

[7] Wang M., Zhang T., Peng H., Luo S., Tan J., Jiang K., Heng Y., Zhang X., Guo X., Zheng J. (2018). Rice *premature leaf senescence 2*, encoding a glycosyltransferase (GT), is involved in leaf senescence. Front. Plant Sci..

[8] Chen L., Wuriyanghan H., Zhang Y., Duan K., Chen H., Li Q., Lu X., He S., Ma B., Zhang W. (2013). An S-domain receptor-like kinase, OsSIK2, confers abiotic stress tolerance and delays dark-induced leaf senescence in rice. Plant Physiol..

[9] Wang Z., Wang Y., Hong X., Hu D., Liu C., Yang J., Li Y., Huang Y., Feng Y., Gong H. (2015). Functional inactivation of UDP-N-acetylglucosamine pyrophosphorylase 1 (UAP1) induces early leaf senescence and defence responses in rice. J. Exp. Bot..

[10] Leng Y., Yang Y., Ren D., Huang L., Dai L., Wang Y., Chen L., Tu Z., Gao Y., Li X. (2017). A rice PECTATE LYASE-like gene is required for plant growth and leaf senescence. Plant Physiol..

[11] Hong Y., Zhang Y., Sinumporn S., Yu N., Zhan X., Shen X., Chen D., Yu P., Wu W., Liu Q. (2018). Premature leaf senescence 3, encoding a methyltransferase, is required for melatonin biosynthesis in rice. Plant J..

[12] Wu L., Ren D., Hu S., Li G., Dong G., Jiang L., Hu X., Zeng D., Qian Q., Guo L. (2016). Down-regulated of a nicotinate phosphoribosyltransferase gene, *OsNaPRT1*, leads to withered leaf tips. Plant Physiol..

[13] Petrov V., Hille J., Mueller-Roeber B., Gechev T.S. (2015). ROS-mediated abiotic stress-induced programmed cell death in plants. Front. Plant Sci..

[14] Saxena I., Srikanth S., Chen Z. (2016). Cross talk between H_2_O_2_ and interacting signal molecules under plant stress response. Front. Plant Sci..

[15] Gill S.S., Tuteja N. (2010). Reactive oxygen species and antioxidant machinery in abiotic stress tolerance in crop plants. Plant Physiol. Biochem..

[16] Hossain M.A., Bhattacharjee S., Armin S.M., Qian P., Xin W., Li H.Y., Burritt D.J., Fujita M., Tran L.S. (2015). Hydrogen peroxide priming modulates abiotic oxidative stress tolerance: Insights from ROS detoxification and scavenging. Front. Plant Sci..

[17] Chen G. (2018). A Library of *japonica* Rice T-DNA Insertion Mutants Was Screened under Oxidative Stress Conditions.

[18] Chen G. (2018). The Phenotype of *RLS1*-RNAi and Transgenic Plants Carrying an Empty Vector at Both Vegetative and Reproductive Stage.

[19] Lim P.O., Kim H.J., Hong G.N. (2007). Leaf senescence. Annu. Rev. Plant Biol..

[20] Zhou Q., Yu Q., Wang Z., Pan Y., Lv W., Zhu L., Chen R., He G. (2013). Knockdown of *GDCH* gene reveals reactive oxygen species-induced leaf senescence in rice. Plant Cell Environ..

[21] Feussner K., Feussner I., Leopold I., Wasternack C. (1997). Isolation of a cDNA coding for an ubiquitin-conjugating enzyme UBC1 of tomato—The first stress-induced UBC of higher plants. FEBS Lett..

[22] Cui F., Liu L., Zhao Q., Zhang Z., Li Q., Lin B., Wu Y., Tang S., Xie Q. (2012). *Arabidopsis* ubiquitin conjugase UBC32 Is an ERAD component that functions in brassinosteroid-mediated salt stress tolerance. Plant Cell.

[23] Zhou G.A., Chang R.Z., Qiu L.J. (2010). Overexpression of soybean ubiquitin-conjugating enzyme gene *GmUBC2* confers enhanced drought and salt tolerance through modulating abiotic stress-responsive gene expression in *Arabidopsis*. Plant Mol. Biol..

[24] Wan X.R., Mo A.Q., Liu S.A., Yang L.X., Li L. (2011). Constitutive expression of a peanut ubiquitin-conjugating enzyme gene in *Arabidopsis* confers improved water-stress tolerance through regulation of stress-responsive gene expression. J. Biosci. Bioeng..

[25] Chung E., Cho C.W., So H.A., Kang J.S., Chung Y.S., Lee J.H. (2013). Overexpression of *VrUBC1*, a mung bean E2 ubiquitin-conjugating enzyme, enhances osmotic stress tolerance in *Arabidopsis*. PLoS ONE.

[26] Bahmani R., Kim D., Lee B.D., Hwang S. (2017). Over-expression of tobacco *UBC1* encoding a ubiquitin-conjugating enzyme increases cadmium tolerance by activating the 20S/26S proteasome and by decreasing Cd accumulation and oxidative stress in tobacco (*Nicotiana tabacum*). Plant Mol. Boil..

[27] Sun L., Wang Y., Liu L.L., Wang C., Gan T., Zhang Z., Wang Y., Wang D., Niu M., Long W. (2017). Isolation and characterization of a *spotted leaf 32* mutant with early leaf senescence and enhanced defense response in rice. Sci. Rep..

[28] He Y., Zhang Z., Li L., Tang S., Wu J.L. (2018). Genetic and Physio-Biochemical Characterization of a Novel Premature Senescence Leaf Mutant in Rice (*Oryza sativa* L.). Int. J. Mol. Sci..

[29] Akhter D., Qin R., Nath U.K., Alamin M., Jin X., Shi C. (2018). The brown midrib leaf (*bml*) mutation in rice (*Oryza sativa* L.) causes premature leaf senescence and the induction of defense responses. Genes.

[30] Bieker S., Riester L., Stahl M., Franzaring J., Zentgraf U. (2012). Senescence-specific alteration of hydrogen peroxide levels in *Arabidopsis thaliana* and oilseed rape spring variety *Brassica napus* L. cv. Mozart. J. Integr. Plant Biol..

[31] Cui M.H., Ok S.H., Yoo K.S., Jung K.W., Yoo S.D., Shin J.S. (2013). An *Arabidopsis* cell growth defect factor-related protein, CRS, promotes plant senescence by increasing the production of hydrogen peroxide. Plant Cell Physiol..

[32] Draper H.H., Hadley M. (1989). Malondialdehyde determination as index of lipid peroxidation. Methods Enzymol..

[33] Shin J.H., Yoshimoto K., Ohsumi Y., Jeon J.S., An G. (2009). *OsATG10b*, an autophagosome component, is needed for cell survival against oxidative stresses in rice. Mol. Cells.

[34] Wu A., Allu A.D., Garapati P., Siddiqui H., Dortay H., Zanor M.I., Asensi-Fabado M.A., Munné-Bosch S., Antonio C., Tohge T. (2012). JUNGBRUNNEN1, a reactive oxygen species-responsive NAC transcription factor, regulates longevity in *Arabidopsis*. Plant Cell.

[35] Cho K., Shibato J., Agrawal G.K., Jung Y.H., Kubo A., Jwa N.S., Tamogami S., Satoh K., Kikuchi S., Higashi T. (2008). Integrated transcriptomics, proteomics, and metabolomics analyses to survey ozone responses in the leaves of rice seedling. J. Proteome Res..

[36] Ramamoorthy R., Jiang S.Y., Kumar N., Venkatesh P.N., Ramachandran S. (2008). A comprehensive transcriptional profiling of the WRKY gene family in rice under various abiotic and phytohormone treatments. Plant Cell Physiol..

[37] Miao Y., Laun T., Zimmermann P., Zentgraf U. (2004). Targets of the WRKY53 transcription factor and its role during leaf senescence in *Arabidopsis*. Plant Mol. Biol..

[38] Liang C., Wang Y., Zhu Y., Tang J., Hu B., Liu L., Ou S., Wu H., Sun X., Chu J. (2014). OsNAP connects abscisic acid and leaf senescence by fine-tuning abscisic acid biosynthesis and directly targeting senescence-associated genes in rice. Proc. Natl. Acad. Sci. USA.

[39] Lai Z., Wang F., Zheng Z., Fan B., Chen Z. (2011). Acritical role of autophagy in plant resistance to necrotrophic fungal pathogens. Plant J..

[40] Li F.Z., Jin S.H., Hu G.C., Fu Y.P., Si H.M., Jiang D.A., Sun Z.X. (2005). Isolation and physiological characteristics of a premature senescence mutant in rice (*Oryza sativa* L.). J. Zhejiang Univ. Sci. B.

[41] Chen G., Feng H., Hu Q., Qu H., Chen A., Yu L., Xu G. (2015). Improving rice tolerance to potassium deficiency by enhancing *OsHAK16p:WOX11*-controlled root development. Plant Biotechnol. J..

[42] Xiong J., Tao T., Luo Z., Yan S., Liu Y., Yu X., Liu G., Xia H., Luo L. (2017). RNA editing responses to oxidative stress between a wild abortive type male-sterile line and its maintainer line. Front. Plant Sci..

[43] Chen G., Zhang Y., Ruan B., Guo L., Zeng D., Gao Z., Zhu L., Hu J., Ren D., Yu L. (2018). OsHAK1 controls the vegetative growth and panicle fertility of rice by its effect on potassium-mediated sugar metabolism. Plant Sci..

[44] Liu Y.G., Mitsukawa N., Oosumi T., Whittier R.F. (1995). Efficient isolation and mapping of *Arabidopsis thaliana* T-DNA insert junctions by thermal asymmetric interlaced PCR. Plant J..

[45] Zhu L., Hu J., Zhu K., Fang Y., Gao Z., He Y., Zhang G., Guo L., Zeng D., Dong G. (2011). Identification and characterization of SHORTENED UPPERMOST INTERNODE 1, a gene negatively regulating uppermost internode elongation in rice. Plant Mol. Biol..

[46] Miki D., Itoh R., Shimamoto K. (2005). RNA silencing of single and multiple members in a gene family of rice. Plant Physiol..

[47] Chen G., Hu Q., Luo L., Yang T., Zhang S., Hu Y., Yu L., Xu G. (2015). Rice potassium transporter OsHAK1 is essential for maintaining potassium-mediated growth and functions in salt tolerance over low and high potassium concentration ranges. Plant Cell Environ..

[48] Chen G., Liu C., Gao Z., Zhang Y., Zhu L., Hu J., Ren D., Xu G., Qian Q. (2018). Driving the expression of *RAA1* with a drought-responsive promoter enhances root growth in rice, its accumulation of potassium and its tolerance to moisture stress. Environ. Exp. Bot..

[49] Liu S., Hua L., Dong S., Chen H., Zhu X., Jiang J.E., Zhang F., Li Y., Fang X., Chen F. (2015). OsMAPK6, a mitogen-activated protein kinase, influences rice grain size and biomass production. Plant J..

[50] Deng L., Qin P., Liu Z., Wang G., Chen W., Tong J., Xiao L., Tu B., Sun Y., Yan W. (2017). Characterization and fine-mapping of a novel premature leaf senescence mutant *yellow leaf and dwarf 1* in rice. Plant Physiol. Biochem..

[51] Chen G., Liu C., Gao Z., Zhang Y., Jiang H., Zhu L., Ren D., Yu L., Xu G., Qian Q. (2017). OsHAK1, a high-affinity potassium transporter, positively regulates responses to drought stress in rice. Front. Plant Sci..

[52] Pan L., Zhang X., Wang J., Ma X., Zhou M., Huang L., Nie G., Wang P., Yang Z., Li J. (2016). Transcriptional profiles of drought-related genes in modulating metabolic processes and antioxidant defenses in *Lolium multiflorum*. Front. Plant Sci..

[53] Chen G., Liu C., Gao Z., Zhang Y., Zhang A., Zhu L., Hu J., Ren D., Yu L., Xu G. (2018). Variation in the abundance of *OsHAK1* transcript underlies the differential salinity tolerance of an *indica* and a *japonica* rice cultivar. Front. Plant Sci..

