# Supplementary material for: Knocking Out the Gene RLS1 Induces Hypersensitivity to Oxidative Stress and Premature Leaf Senescence in Rice
Source: Int J Mol Sci. 2018 Sep 20;19(10):2853. doi: 10.3390/ijms19102853 (PMC6213272; doi:10.3390/ijms19102853)
Supplement: Supplementary file 1 [file ijms-19-02853-s001.zip › ijms-359672 supplementary for final check.docx]

**Table S1.** Primer sequences used for mapping *RLS1* and sequence analysis.

| Primers (5’ to 3’) Sense | Anti-sense | Length (bp) |
| --- | --- | --- |
| E41F: 5'-GCCTGTAACTTCTAATGGG-3' | E41R: 5'-ATGGGAAATG CTATCACAAT-3' | 185 |
| E42F: 5'-CTGGGGACATTAGCCTTTC-3' | E42R: 5'-TGCTTTTGTT CCTTGAGTA-3' | 250 |
| E43F: 5'-TGTAAATCGATAGTTTTGAG-3' | E43R: 5'-TATGGTGTAG TAGGAAGAG-3' | 157 |
| E45F: 5'-TTCACCTTCCCCATGAAAC-3' | E45R: 5'-TACTCCCTCT GTCCCTTAA-3' | 197 |
| E47F: 5'-ATGGGTCTCATTCCGTTCTA-3' | E47R: 5'-TTATCCGCAT CAGCCAAAGT-3' | 205 |
| E48F: 5'-ATGCTCCGAATCCGACACCT-3' | E48R: 5'-AGCCTGCAAC CTCGTGAGTG-3' | 241 |
| E49F: 5'-ACGCTCTTTGGAATTTTCAT-3' | E49R: 5'-GTCTATTTTG GGTCCCTCAG-3' | 199 |
| E50F: 5'-GAACCTTTTCTTTTACTGA-3' | E50R: 5'-CTAAAATGAT CCATTCCAT-3' | 185 |
| E54F: 5'-ATTTCGGAGGGAGTTTGTAT-3' | E54R: 5'-AACCTGGATTATTGGGATGT-3' | 231 |
| E55F: 5'-TATCCTAAAAGCAGCACAAT-3' | E55R: 5'-AGTAGCATGGTCAGCACTAC-3' | 139 |
| E58F: 5'-ACAAGGAAACATCTGAAAGTC-3' | E58R: 5'-TCAATCAAGAAGGCACCCAAA-3' | 191 |
| E59F: 5'-ATAGATGATTGGTAGCAGGAA-3' | E59R: 5'-GTAAAGTAAGGGAAAATTGGA-3' | 181 |
| E60F: 5'-CGCTTCCAGTGTTTGCTTGTG-3' | E60R: 5'-ACAACCTCCAAGAAATATGCA-3' | 189 |
| E62F: 5'-TTGTAGGGGATGTTTTGGTG-3' | E62R: 5'-GCCTTGAAATCGTAATGGAA-3' | 227 |
| E52F: 5'-CACAGCATGAATCACCTATA-3' | E52R: 5'-AGACATGATG TGGCTTACTC-3' | 245 |
| E21F: 5'-TCCCAAACTACTAAATGGTACG-3' | E21R: 5'-GTCTACGAAAAATGAAGCAAAT-3' | 221 |
| E31F: 5'-TGTTCATCACCAGGCATTG-3' | E31R: 5'-CGGTGCTGAACTATTGCTG-3' | 200 |
| RM31F: 5'-GATCACGATCCACTGGAGCT-3' | RM31R: 5'-AAGTCCATTACTCTCCTCCC-3' | 147 |
| E33F: 5'-TAATAAGACGAATAGTCAAACAG-3' | E33R: 5'-GAGGAGGACATCAAGCACAT-3' | 179 |
| E35F: 5'-CTCGATTATCGTGGAAGAAGAG-3' | E35R: 5'-AAAGGGCAGTTACGGAAGC-3' | 117 |
| E36F: 5'-CAGCAGCAGCAGGCAGAGGA-3' | E36R: 5'-GTCACGCACGGAACGAGGA-3' | 121 |
| FE14P1: 5’-AATAATCTGAACCATAAGTTCCGATAGC-3’ | FE14P22: 5’-GGATTTCTGCATGGAGACATAGTT-3’ | 705 |
| ls4F: 5’-AGTCCGCTGGCATCTACCATT-3’ |  |  |
| ls41F: 5’-CACGGTTCCATTGGTAAGATTTGCT-3’ |  |  |
| ls42F: 5’-CAGGGACCGATCCATGTTTCA -3’ |  |  |
| ls4R: 5’-TGGGCCGTGTAGTTGGAGTTG-3’ |  |  |
| ls421R: 5’- AGCCCATTCCTCCAATCCTTT-3’ |  |  |
| ls422R: 5’- TTCGGATTGCGAGGGAAGTCT-3’ |  |  |

**Table S2.** Primer sequences used for qRT-PCR assays.

| Gene | Primer ID | Primer sequences | |
| --- | --- | --- | --- |
| *UBQ5* | F(5’-3’) | | CTCGCCGACTACAACATCCA |
|  | R(5’-3’) | | TCTTGGGCTTGGTGTACGTCTT |
| *SGR* | F(5’-3’) | | GCAATGTCGCCAAATGACG |
|  | R(5’-3’) | | GCTCACCACACTCATTCCCTAAAG |
| *Osl2* | F(5’-3’) | | GCAGACAACAAATCGCCAAAT |
|  | R(5’-3’) | | TCTCCAGCAACTCTAACCAGCAT |
| *Osl20* | F(5’-3’) | | GCGGCACAAGTGAGGGAGAC |
|  | R(5’-3’) | | TTGGGGTGCTGATTGTCCAG |
| *Osl30* | F(5’-3’) | | AACCTTTTTCTTGGAGATGATACAA |
|  | R(5’-3’) | | CTTGAACTGTAGGGGCTT |
| *Osl43* | F(5’-3’) | | TGTGACAAGTGCTAATAATACATACGA |
|  | R(5’-3’) | | CCAGACCTTCCAAAGAATCCAAC |
| *Osl85* | F(5’-3’) | | ACGCTGCAGCACCAGAAGTG |
|  | R(5’-3’) | | CACTTCCAGGCCCTGTCCAT |
| *Osh36* | F(5’-3’) | | TGCCCGGCTTTACACTGGTT |
|  | R(5’-3’) | | GCGGTAGCACCCATTGTTCC |
| *Osh69* | F(5’-3’) | | TGCCGGAGCAAGAACTCTAC |
|  | R(5’-3’) | | GGGATTCAGCAAGAACCTGA |
| *NYC1* | F(5’-3’) | | CATGCAACACCAACAAAAGG |
|  | R(5’-3’) | | GACCATTCCAGGAGAAGCAG |
| *NYC3* | F(5’-3’) | | TGTCGTTGCCATGTGAAGAT |
|  | R(5’-3’) | | TTGGTCACGCCACAAATCTA |
| *OsWRKY23* | F(5’-3’) | | TCCAGTTCCTCTCCCAGTTCTAA |
|  | R(5’-3’) | | CACATTGTTCTCCTTTTCTTCCC |
| *OsWRKY72* | F(5’-3’) | | CACCACAAATCACATCTACTCCG |
|  | R(5’-3’) | | GCTGAAGGGAAGAGAGGTGAG |
| *OsNAC2* | F(5’-3’) | | AAAAACAACCGCATTGGCAG |
|  | R(5’-3’) | | AGTCCTCATCTCCTCTGTCTAATCC |
| *OsATG3b* | F(5’-3’) | | CGAGCAAGAGGAAGCCGTAT |
|  | R(5’-3’) | | CGTCTTACGCAAGGCACGTT |
| *OsATG7* | F(5’-3’) | | GTGATGCAGGTGATCAACGAA |
|  | R(5’-3’) | | GCCGCTGATTTCATCAAGTCA |
| *OsATG8a* | F(5’-3’) | | AGCCCAGAAAAGGCCATCTT |
|  | R(5’-3’) | | CATCCTTGTTCTCTTCGTAGATTGC |
| *OsATG8b* | F(5’-3’) | | ATTCCTGACATCGACAAGAAAAAGT |
|  | R(5’-3’) | | CGCTTCCGAACAACATAGACAA |
| *OsATG8c* | F(5’-3’) | | TCGTCAAGAACACGCTTCCA |
|  | R(5’-3’) | | GCCGTCCTCATCCTTGTTCTC |
| *OsATG10b* | F(5’-3’) | | GGACATTTATTACTCGGGAGTTGAA |
|  | R(5’-3’) | | TGAACCATGGCCTACTAAAATGG |
| *OsATG12* | F(5’-3’) | | CCGCCGAGCAGAAGAAAG |
|  | R(5’-3’) | | TTCCACCAATCTTGAACTTGGA |
| *OsATG13a* | F(5’-3’) | | AAGGAGGTTGTGTGGTAAGAGATGT |
|  | R(5’-3’) | | TAACAAGCGCCGGTTGACA |
| *OsATG13b* | F(5’-3’) | | GCAGTGATGGGAAGGAAGCTA |
|  | R(5’-3’) | | GAGCCAACTGCGGCATCTT |
